# Single-centre comparison of non-familial, familial and monogenic lupus

**DOI:** 10.1093/rheumatology/keaf304

**Published:** 2025-06-04

**Authors:** Jalila Alshekaili, Batool S H Al Lawati, Zahran Althuhli, Warda Al-Azri, Almundher Al-Maawali, Samiya Al-Rashdi, Farida Al-Mamari, Mahmood Al-Kindi, Hamad Al Balushi, Mohammed Al-Rawahi, Murtadha Al-Khabori, Matthew C Cook

**Affiliations:** Department of Microbiology and Immunology, Sultan Qaboos University Hospital, University Medical City, Muscat, Oman; Rheumatology Unit, Department of Medicine, College of Medicine and Health Sciences, Sultan Qaboos University, Muscat, Oman; College of Medicine and Health Sciences, Sultan Qaboos University, Muscat, Oman; Rheumatology Unit, Department of Medicine, Sultan Qaboos University Hospital, University Medical City, Muscat, Oman; Department of Genetics, College of Medicine and Health Sciences, Sultan Qaboos University, Muscat, Oman; Department of Genetics, Sultan Qaboos University Hospital, University Medical City, Muscat, Oman; Department of Genetics, Sultan Qaboos University Hospital, University Medical City, Muscat, Oman; Department of Genetics, College of Medicine and Health Sciences, Sultan Qaboos University, Muscat, Oman; Department of Microbiology and Immunology, Sultan Qaboos University Hospital, University Medical City, Muscat, Oman; Department of Microbiology and Immunology, Sultan Qaboos University Hospital, University Medical City, Muscat, Oman; Department of Hematology, College of Medicine and Health Sciences, Sultan Qaboos University, Muscat, Oman; Department of Hematology, College of Medicine and Health Sciences, Sultan Qaboos University, Muscat, Oman; Department of Immunology and Infectious Disease, John Curtin School of Medical Research, Australian National University, Canberra, NSW, Australia; Centre for Personalised Immunology (NHMRC Centre of Research Excellence), John Curtin School of Medical Research, Australian National University, Canberra, NSW, Australia; Cambridge Institute for Therapeutic Immunology and Infectious Disease, Department of Medicine, University of Cambridge, Cambridge, UK

**Keywords:** systemic lupus erythematosus (SLE), monogenic lupus, IKZF2, DNASE1L3, COPA

## Abstract

**Objectives:**

There is a significant genetic contribution to systemic lupus erythematosus (SLE). Monogenic SLE is a distinct form of SLE. We investigated whether familial or known monogenic lupus cases are distinguishable from non-familial lupus by clinical or laboratory phenotype in a population with high rates of consanguinity.

**Patients and methods:**

We performed a retrospective case-control study on 24 multiplex families, comprising 62 cases, and 95 non-familial lupus controls. Demographic and Systemic Lupus International Collaborating Clinics criteria were compared between the two groups. Familial cases were also evaluated by whole exome sequencing and cellular phenotyping. Statistical analysis was performed using R Studio.

**Results:**

Familial and non-familial lupus cases were similar although familial cases were younger at presentation (18 y *vs* 26 y, OR = 0.91, *P = *1.61 × 10^−5^), a higher prevalence of synovitis (OR = 2.7, *P = *0.013) and lower prevalence of high level of dsDNA antibodies (OR = 0.25, *P = *1.9 × 10^−3^). Exome sequencing of a subset yielded a diagnostic rate of 36%. Monogenic lupus were distinguished from other familial cases by an even younger age at presentation (6 y *vs* 19 y, OR = 0.82, *P = *2.13 × 10^−3^), non-biased male-to-female ratio (*P = *0.077) and expansion of exhausted CD4^+^ T cells (CD4+CD45RA+PD-1+) (*P = *1.7 × 10^−4^).

**Conclusion:**

Overall, clinical phenotype is a poor indicator of familial or monogenic lupus. Familial lupus and monogenic familial lupus (MoFL) tend to present at a younger age than the Non-MoFL, exhibit less female bias and in some cases are distinguished by a lymphocyte signature. Consanguinity increases the rate of monogenic familial lupus and these cases can present in adulthood.

Rheumatology key messages

- Younger age and cellular phenotypes but not clinical manifestations distinguish familial and monogenic SLE.
- Normalization of male-to-female ratio is seen in monogenic SLE.
- WES is probably indicated in lupus cohorts drawn from populations with high rates of consanguinity irrespective of age of presentation.

## Introduction

Systemic lupus erythematosus (SLE) is a chronic autoimmune disease with a significant genetic contribution to its aetiology. Monozygotic (MZ) twin concordance for SLE has been reported to range from 11% to 40% compared with zero to 4% for dizygotic (DZ) twins [1–3]. In another study, probandwise concordance rates were 14.3% and 7.7% among MZ and DZ twins, respectively [4]. Furthermore, relatives of SLE patients are at a significantly higher risk of developing SLE [relative risk for siblings, 23.68 (20.13–27.84)] as well as other autoimmune diseases [5].

While the genetic contribution to lupus is usually attributed to an aggregation of weak acting risk alleles, many monogenic causes of lupus have now been identified (Supplementary Table S1, available at *Rheumatology* online) [6–13]. While monogenic lupus is rare, it is an important subset because of its potential to yield pathogenic insights. It is difficult to determine disease mechanisms in polygenic disease, whereas analysis of monogenic lupus has proved highly informative.

Monogenic cases are sometimes associated with distinct clinical manifestations. Patients with complement deficiencies present with frequent infections, rashes but absence of dsDNA antibodies [7, 8, 14]. DNASE1L3 deficiency presents with renal lupus, hypocomplementemia urticarial vasculitis and positive anti-neutrophil cytoplasmic antibodies (ANCA) [6, 9–11]. Interferonopathies conferred by defects in RNASEH2A/B/C, TREX1, DNASE2 are often characterized by neurological manifestations and cutaneous vasculitis or chilblains lupus [6, 10, 11].

Identification of patients with monogenic lupus is a priority, both for affected patients and their families, and for advancing our understanding of lupus. Of course, genotype-first approaches provide a definitive method to ascertainment of such cases but this is not always practical. It remains unclear how to best stratify patients for sequencing in order to identify monogenic cases within general lupus clinics. To date, comparison of familial and non-familial European lupus cohorts has revealed considerable similarities between the two groups [15, 16]. One interpretation of these findings is that familial cases and non-familial cases arise from a similar burden of common lupus-associated alleles. However, as the prevalence of monogenic lupus is very low, it remains unclear from these studies whether such cases could be identified within a larger cohort of familial and non-familial lupus.

Here we report on a case-control study of familial and non-familial lupus in a population with a high rate of consanguinity from which we expect a higher rate of monogenic disease within the familial cohort than has been observed previously. We aimed to assess whether familial known monogenic lupus cases and non-familial SLE are distinguishable from each other by any of the known clinical or laboratory phenotypes in a population with high rates of consanguinity.

## Patients and methods

### Human subjects

We performed a single-centre retrospective case-control study to investigate the demographics, clinical and laboratory features of familial and non-familial lupus. All cases were recruited from the adult rheumatology clinic at the Sultan Qaboos University Hospital (SQUH). Familial lupus was defined as any case with one or more affected family members (sibling, offspring, uncle, aunt, first-degree cousin and second-degree cousin). Non-familial cases were defined as those in which no other family member was affected with SLE at the time of recruitment. No *a priori* power estimates were performed. We aimed to collect all available familial lupus cases during the study period. In an effort to increase statistical power and identify a meaningful effect, we recruited familial and non-familial cases in a ratio of 1:1.5. We randomly selected 95 non-familial cases from a list of 400 lupus patients identified only by medical record numbers and initials after excluding familial cases.

The study was approved by the medical research ethic committee of the College of Medicine and Health Sciences, Sultan Qaboos University, Sultanate of Oman (MREC#2003 and MREC#1859). Informed written consent was obtained from all participants or their guardians. All methods were performed in accordance with the relevant guidelines and regulations and in accordance with the declaration of Helsinki.

### Data collection

Lupus was defined and assessed according to SLICC criteria [17]. Data were collected from the SQUH Information System (Track Care) and included 11 clinical and six immunological criteria as well as demographic characteristics: current age, age at diagnosis, and sex. Eleven clinical and six immunological SLICC criteria were included for final analysis. The clinical criteria were acute cutaneous lupus, chronic cutaneous lupus, oral ulcers, a non-scarring alopecia, synovitis, serositis, renal including renal biopsy, neurologic, hemolytic anaemia, leukopenia (or lymphopenia) and thrombocytopenia. ANA, anti-dsDNA (twice the level of the normal range detected by ELISA), anti-Smith, antiphospholipid antibody and low complement were determined. These criteria were counted cumulatively and did not have to be present concurrently to be scored.

### Whole exome sequencing and variant interpretation

Familial cases with one or more member with childhood onset were offered whole exome sequencing (WES), which was performed in-house or commercially. In brief, genomic DNA of the probands and family members were isolated from peripheral blood using a DNeasy Blood and Tissue Kit (Qiagen, Courtaboeuf, France). Later, DNA was barcoded and enriched for the coding exons of targeted genes using hybrid capture technology (Agilent SureSelect Human All-exons-V7, CA, USA). Prepared DNA libraries were then sequenced using a Next-Generation Sequencing (NGS) Illumina technology (NovaSeq6000, CA, USA) of 150 bp paired-end, at 150–200× coverage. The reads were mapped against UCSC GRCh37/hg19 or GRCh38/hg38 by Burrows-Wheeler Aligner (BWA 0.7.12). Genome Analysis Tool Kit (GATK v3.4 or Haplotypecaller in GATK v4) was used for variant calling.

Raw data were analysed using the in-house annotation and analysis pipelines. Collected raw data (BAM and VCF files) were pre-processed using BCFtools (v.1.5), and then annotation was completed using ANNOVAR tools (version release 2015 Dec14 with its updates). Left alignment and decomposition of multiallelic sites were completed for all raw files using the same pipeline. Allele frequency was determined by reference to available variant databases (1000 Genomes, Exome Variant Server and GnomAD) as well as an in-house database of 1564 Omani exomes. VCF files were screened for rare variants of genes known to cause monogenic lupus (Supplementary Table S1, available at *Rheumatology* online). Cascade testing for monogenic causes was performed by Sanger sequencing using primers listed in Supplementary Table S2, available at *Rheumatology* online.

### Lymphocyte phenotyping

Cellular T-cell and B-cell phenotyping was performed by flow cytometry. One hundred microlitres of blood was added to the desired cocktail of antibodies and incubated for 20 min at room temperature. Lysing solution optilyse-B was then added according to the manufacture recommendations. This was followed up with a wash step and then acquisition of the sample using Navios flow cytometer (Brea, CA, USA). Data analysis was done using Flow-Jo software.

The following antibodies were used: anti-CD45-Krome Orange (clone J33), anti-CD19-ECD (clone J3119), anti-CD21-PE (clone BL13), anti-CD27-PC7 (clone 1A4CD27), and anti-CD38-APC-A750 (clone LS198-4–3), anti-IgD-FITC (clone IA6-2), anti-IgM-PB (clone SA-DA4) and anti-CD24-APC (clone ALB9), as Dura clone IM B cells. Anti-CD45-Krome Orange (clone J33), anti-CD3-APC-A750 (clone UCHT-1), anti-CD4-APC (clone 13B8.2), antiCD8-A700 (clone B9.11), anti-CD45RA-FITC (clone 2H4), anti-CCR7-PE (clone G043H7), anti-CD28-ECD (cloneCD28.2), anti-PD-1-PC5.5 (clone PD13.5), anti-CD27-PC7(clone1A4.CD27), anti-CD57-PB (NC1) as Dura clone IM T-cell subsets, all from Beckman Coulter.

### Statistical analysis

Clinical and laboratory data were extracted from the electronic medical record and transferred into excel and further analysed using RStudio [18]. Descriptive statistics were used to summarize continuous and categorical variables as appropriate. Results were expressed as medians with their interquartile ranges (IQR) and frequency (%) for continuous and categorical variables, respectively. Collected demographic data and clinical and immunological criteria were compared between the familial and non-familial groups using univariate logistic regression. Variables with *P ≤ *0.10 were further entered into a multivariate logistic regression model to examine their independent association with familial lupus. A similar approach of univariate and multivariate logistic regression was used to assess the differences between the monogenic familial group *vs* non monogenic familial group. *P*-value of <0.05 was used for statistically significant. Lymphocytes phenotypic examination was done and analysis for the cellular differences between the different examined monogenic groups was done using ANOVA. To control for the increased likelihood of Type I errors due to multiple comparisons, we applied the false discovery rate (FDR) correction using the Benjamini–Hochberg method. *P*-values were calculated to assess the statistical significance of the results. *P < *0.05 was considered as significant after adjusting for multiple tests using FDR correction.

## Results

### Familial cases

We identified 24 families in which there was more than one member with SLE. All affected members (*n* = 62) were included in the study. For comparison, we identified 95 non-familial cases of SLE (as described in the Methods). Concordance for lupus approximated genetic relatedness (Table 1). Among the 24 lupus families, 50% of the affected cases were sibling pairs (estimated shared genome ∼50%), followed by affected first cousins (20.8%) (estimated shared genome ∼12.5%). Affected aunt/uncle and mother-offspring accounted for 16.7% and 12.5%, respectively (estimated shared genome ∼25%).

**Table 1.** Relationships of affected members of multiplex families

Relations of affects	Approximate genome sharing	Number of families (%)	Number of affected (%)
Sibling pair	50%	12/24 (50.0%)	25/62 (40.3%)
Mother/daughter pair	50%	3/24 (12.5%)	7/62 (11.1%)
Aunt/uncle/niece pair	25%	4/24 (16.7)	12/62 (19.0%)
Cousin/cousin pair	12.5%	5/24 (20.8%)	18/62 (28.6%)
Total		24	62

### Demographic characteristics of familial and non-familial cases

Familial cases (*n* = 62) comprised 52 females and 10 males, yielding a female-to-male ratio of 5.2:1 (Table 2). There was a similar female-to-male ratio among the non-familial cases (6.9:1, *P = *0.57). Median age of onset was significantly younger in familial cases (18.0 years) compared with the non-familial cases (26.0 years) (*P* *<* 0.05) and familial cases had a longer disease duration at the time of study recruitment (*P* *<* 0.05).

**Table 2.** Demographic summary of patient cohort.

	Familial (*n* = 62)	Non-familial (*n* = 95)	OR	95% CI	*P*-value
Male	10 (16.1%)	12 (12.6%)			0.54
Female	52 (83.9%)	83 (87.4%)			
Female : male	5.2:1	6.9:1			
Median age in years at presentation (IQ range)	18 (10.25–21.0)	26.0 (20.0–32.0)	0.92	0.88–0.95	7.42 × 10^-6^
Median age in years at study (IQ range)	29.0 (24.0–35.8)	35.0 (26.0–40.0)	0.97	0.93–0.99	0.034
Median disease duration in years (IQ range)	12 (7.25–17.0)	8.0 (5.0–9.0)	1.22	1.13–1.33	1.15 × 10^-6^

### Clinical and immunological phenotypes for the familial and non-familial SLE

We compared the prevalence of clinical and immunological manifestations of SLE in both groups (Table 3). Overall, the most common clinical manifestations were hematological cytopenias followed by synovitis. The prevalence of lymphopenia, leukopenia and synovitis was 77.4%, 67.7% and 56.4%, respectively, among familial lupus. These were also the most common clinical manifestations among non-familial lupus cases (leukopenia, 80.0%; lymphopenia, 75.8%; synovitis, 30.5%).

**Table 3.** Prevalence of SLICC criteria among familial and non-familial SLE groups

			UPR^a^		MPR^b^	
	Familial n = 62	Non-familial n = 95	OR (95% CI)	*P*-value	OR (95% CI)	*P*-value
Median age at presentation (IQ range)	18 (10.25–21.0)	26.0 (20.0–32.0)	0.92 (0.88–0.95)	7.4 × 10^-6^	0.91 (0.87–0.9)	1.6 × 10^-5^
Male (%)	10 (16.1%)	12 (12.6%)		0.54		
Clinical criteria, *n* (%)						
Acute cutaneous lupus	21 (33.9)	22 (23.2)	1.70 (0.83–3.5)	0.14		
Chronic cutaneous lupus	2 (3.2)	0 (0)	NE	0.99		
Ulcers	5 (8.1)	4 (4.2)	1.99 (0.51–8.36)	0.32		
Nonscarring alopecia	7 (11.3)	8 (8.4)	1.38 (0.46–4.07)	0.55		
Synovitis	35 (56.4)	29 (30.5)	2.95 (1.53–5.80)	1.4 × 10^-3^	2.7 (1.2–5.9)	0.013
Serositis	7 (11.3)	4 (4.2)	2.89 (0.84–11.5)	0.10		
Renal criteria with biopsy	24 (38.7)	29 (30.5)	1.44 (0.73–2.82)	0.29		
Neurologic	3 (4.8)	5 (5.3)	0.92 (0.18–3.87)	0.91		
Haemolytic anaemia	14 (22.6)	14 (14.7)	1.69 (0.73–3.87)	0.21		
Leukopenia	42 (67.7)	76 (80.0)	0.53 (0.25–1.09)	0.085		
Lymphopenia	48 (77.4)	72 (75.8)	1.10 (0.52–2.39)	0.81		
Lymphopenia OR Leukopenia	54 (87.1)	90 (94.7)	0.37 (0.11–1.18)	0.10	0.23 (0.05–0.91)	0.041
Thrombo-cytopenia	13 (21.1)	23 (24.2)	0.83 (0.38–1.78)	0.64		
Immunological criteria, *n* (%)						
ANA above laboratory reference range	61 (98.4)	95 (100)	NE	0.99		
Anti-dsDNA twice the range	40 (64.5)	76 (80.0)	0.45 (0.22–0.93)	0.033	0.25 (0.10–0.59)	1.9 × 10^-3^
Anti-Smith antibody	15 (24.2)	34 (35.8)	0.57 (0.27–1.16)	0.127		
Anti-phospholipid antibody	20/54 (37.0)	34/88 (38.7)	0.93 (0.46–1.87)	0.85		
Low complement	52 (83.9)	77 (81.1)	1.22 (0.53–2.93)	0.65		

a Uniparameter regression.

b Multiparameter regression; NE, not estimable due to extreme values resulting from the small sample size.

The least common manifestations for both groups were chronic cutaneous lupus, mouth ulcers and neurological manifestations. The most common immunological manifestations for both groups were hypocomplementemia and high level of double-stranded DNA antibodies (83.9% and 64.5% for familial lupus and 81.1% and 80.0% for the non-familial lupus) (Table 3).

### Differences between familial and non-familial SLE

Clinical and immunological parameters used in the SLICC criteria were examined for any association with familial SLE. Univariate analysis identified age at presentation, synovitis and level of dsDNA antibodies in association with familial lupus (all with *P* *<* 0.05) (Table 3). Multivariate analysis confirmed the association between familial lupus and younger median age at presentation (OR = 0.91, *P *= 1.61 × 10^−5^) and synovitis (OR = 2.7, *P = *0.013), while significantly fewer familial cases had dsDNA antibodies (OR = 0.24, *P* = 1.9 × 10^−3^) (Table 3).

### Monogenic lupus cases

**Figure 1.**
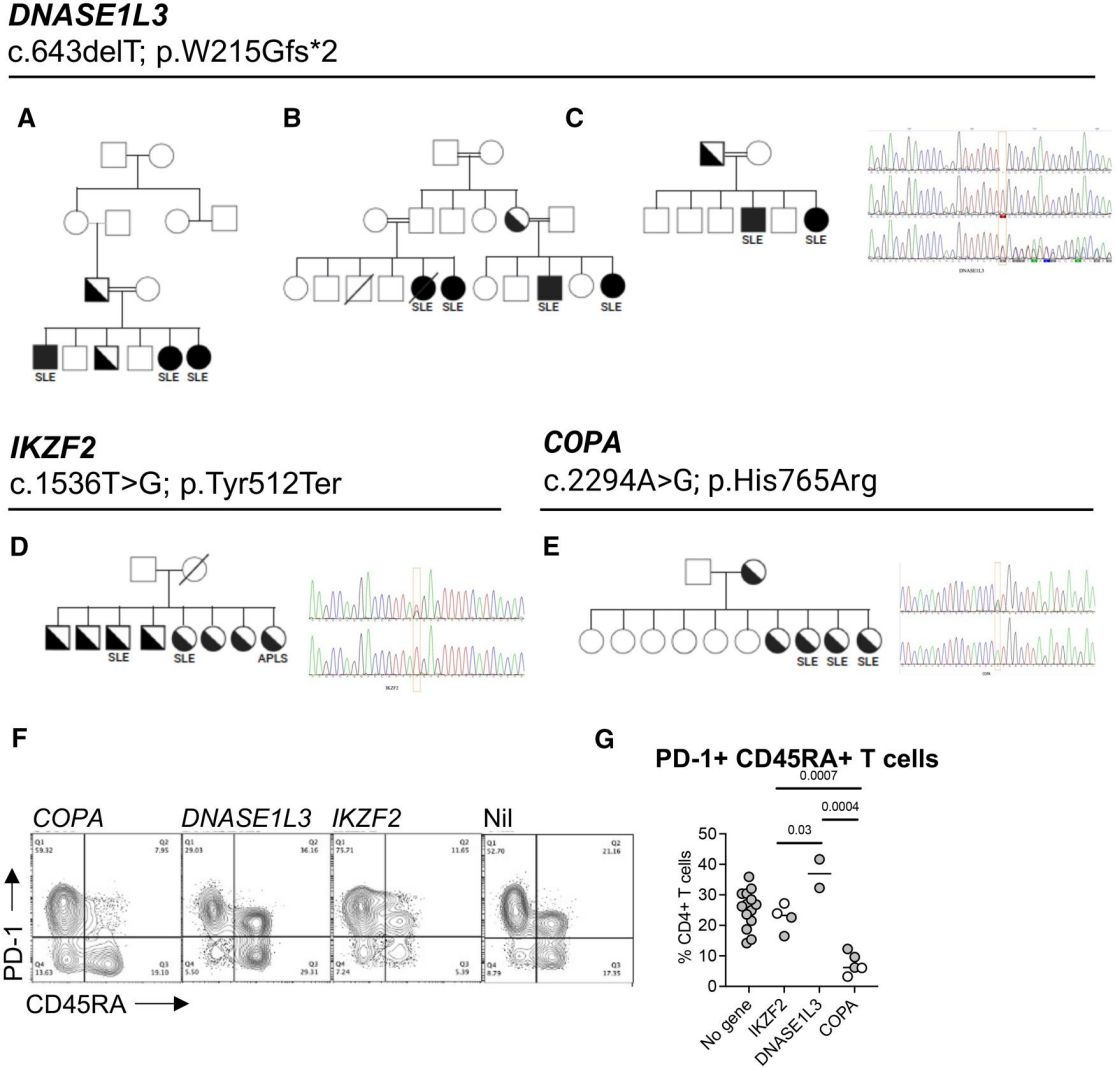
Summary of monogenic lupus cases. Kindred structures of monogenic lupus cases due to variants in *DNASE1L3* (**A**–**C**), *IKZF2* (**D**) and *COPA* (**E**); representative electropherograms of each variant are shown (**F**). Flow cytometric analysis of monogenic lupus cases showing CD4+ T-cell subsets according to CD45RA, PD-1 expression plus summary plots of CD45RA+PD-1+. Each symbol represents the result from a single individual

Fourteen of the 24 lupus families (*n* = 31 lupus patients) underwent whole exome sequencing (WES). Although the exome accounts for only 1–2% of the human genome, ∼85% of variants identified in patients with Mendelian disease are present in the coding genome [19]. Short-read WES is highly sensitive for identifying rare single nucleotide variants, small insertions and deletions and splice-site variants in the coding genome. We identified five families (comprising 11 patients) with likely or confirmed monogenic lupus (monogenic familial lupus, MoFL). Thus, we obtained a diagnostic yield of 36% from WES among recruited families and 35% for affected individuals. Monogenic diagnoses were accounted for by three variants (Fig. 1A–E, Table 4, Supplementary Data S1, available at *Rheumatology* online). Three families containing seven lupus patients were identified with autosomal recessive lupus due to a homozygous frameshift variant in *DNASE1L3*, a lupus-associated variant known to have high prevalence in Oman [20]. All patients with *DNASE1L3* were members of consanguineous families.

**Table 4.** Summary of monogenic lupus genotypes

Gene	Protein	c.DNA	Effect	MAF (global)	MAF (Oman)	ACMG	Ref
*IKZF2*	Tyr512Ter	1536T>G	Premature stop	absent	absent	Likely pathogenic	
*DNASE1L3*	Trp215GlyfsTer2	643del	Frameshift	absent	0.0028	Pathogenic	[20]
*COPA*	His765Arg	2294A>G	Missense	absent	0.00032	Uncertain significance	

We identified a novel *IKZF2* variant (*n* = 2) that is predicted to be a loss-of-function due to the introduction of a premature stop codon. One patient had SLE and the other had primary antiphospholipid syndrome. Cascade testing of the same kindred revealed that the five remaining siblings were also heterozygous for the *IKZF2* variant, one of whom had lupus. Four were clinically healthy although three of them were seropositive for ANA but negative ENA and dsDNA antibodies. Interestingly, the two SLE and one primary APLS patient with *IKZF2* variants had moderate to high levels of anti-phospholipid antibodies while the other siblings, who are clinically healthy, were all negative for anti-phospholipid antibodies. The father did not carry this mutation and eight out of eight siblings were heterozygotes **(**Fig. 1D**)**; thus, the mother was likely to be homozygous for the *IKZF2* variant but she is deceased and not available for testing. At least one previous case has been reported of a heterozygous loss of function variant in *IKZF2* presenting with lupus and incomplete penetrance [21]. Finally, we identified kindred with an ultra-rare heterozygous *COPA* missense variant (*n* = 2). A further three carriers were identified on cascade testing by Sanger sequencing. This variant has not been described previously and is predicted to be damaging. The identified *COPA* carriers included one patient who presented recently with lupus nephritis, and one clinically healthy sibling who was seropositive for ANA but tested negative for antibodies to ENA, dsDNA and APLS. The variant was transmitted from the healthy, seronegative mother (Fig. 1E, Supplementary Data S1, available at *Rheumatology* online).

### Clinical and immunological phenotypes for the MoFL and NonMoFL

Similar to the familial and non-familial SLE, MoFL and non-monogenic familial lupus (non-MoFL) cases had a predominance of hematological manifestations (Table 5). Interestingly, no patient with MoFL was identified with chronic cutaneous lupus, oral ulcers, non-scaring alopecia or neurologic manifestations. Indeed, these manifestations were the least prevalent among the familial group with no genetic diagnosis. The prevalence of other clinical and laboratory parameters was similar in the two groups.

**Table 5.** Prevalence of SLICC clinical and immunological manifestations in familial lupus with or without a genetic diagnosis

	Familial lupus (FL)		UPR^a^		MPR^b^	
	NonMoFL (*n* = 51)	MoFL (*n* = 12)	OR (95% CI)	*P*-value	OR (95% CI)	*P*-value
Median age at presentation in years (IQ range)	19.0 (14.0–23.0)	6 (4–12)	1.20 (1.09–1.38)	1.76 × 10^-3^	0.82 (0.71–0.92)	2.13 × 10^-3^
Male : female (% male)	6:51 (11.8%)	4:12 (33.3%)	0.23 (0.052–1.09)	0.056	5.66 (0.85–45.0)	0.077
Clinical criteria, *n* (%)						
Acute cutaneous lupus	16 (31.4)	5 (45.5)	0.55 (0.14–2.15)	0.38		
Chronic cutaneous lupus	2 (3.9)	0 (0)	NE	0.99		
Aphthous ulcers	5 (7.8)	0 (0)	NE	0.99		
Nonscarring alopecia	7 (13.7)	0 (0)	NE	0.99		
Synovitis	27 (52.9)	8 (72.7)	0.42 (0.08–1.65)	0.24		
Serositis	5 (9.8)	2 (18.2)	0.49 (0.089–3.78)	0.43		
Renal criteria with biopsy	21 (41.2)	3 (27.3)	1.87 (0.48–9.28)	0.40		
Neurologic	3 (5.9)	0 (0)	NE	0.99		
Haemolytic anaemia	12 (23.5)	2 (18.2)	1.38 (0.30–9.90)	0.70		
Leukopenia	33 (64.7)	9 (75.0)	0.41 (0.06–1.80)	0.28		
Lymphopenia	41 (80.4)	7 (63.6)	2.34 (0.53–9.46)	0.24		
Lymphopenia and leukopenia	43 (84.3)	11 (100)	NE	0.99		
Thrombocytopenia	10 (20.0)	3 (27.3)	0.65 (0.15–3.37)	0.57		
Immunological criteria, *n* (%)						
ANA above laboratory reference range	50/51 (98.4)	11 (100)	NE	0.99		
Anti-dsDNA twice the range	32 (62.7)	8 (72.7)	0.63 (0.13–2.49)	0.53		
Anti-Smith antibody	13 (25.4)	2 (18.2)	1.54 (0.34–10.96)	0.61		
Antiphospholipid antibody	16/43 (16.6)	4 (36.4)	1.04 (0.27–4.47)	0.96		
Low complement	41 (80.3)	11 (100)	NE	0.99		

a Uniparameter regression.

b Multiparameter regression; NE, not estimable due to extreme values resulting from the small sample size.

### Differences of clinical and immunological phenotypes between MoFL and NonMoFL

We compared MoFL with non-monogenic familial lupus (NonMoFL) cases and observed a possible reduction in female bias in the monogenic group compared with the non-monogenic group (*P = *0.056; multivariate analysis, *P = *0.077) (Table 5). Median age at presentation was significantly younger for MoFL cases than NonMoFL cases (MoFL: 6 years, *vs* NonMoFL, 19 years, *P *= 2.13 × 10^−3^). There were no other significant differences between the two groups for clinical and immunological parameters (Table 5).

### Cellular alteration associated with the involved gene

Finally, we compared abundance of different T-cell and B-cell subsets in MoFL (*n* = 6), NonMoFL (*n* = 14) and unaffected carriers of monogenic variants (*n* = 5). We observed a substantial increase in exhausted CD4+ T cells (CD3+ CD4+ PD-1+ CD45RA+) (*P *= 1.74 × 10^−4^) (Supplementary Table S3, available at *Rheumatology* online). This difference was accounted for by patients with the *DNASE1L3* variant where 37% of CD4+ T cells were identified with this phenotype (PD-1+ CD45RA+) (Fig. 1F).

## Discussion

Overall, we found that although familial and non-familial lupus cases were similar in clinical and laboratory manifestations, a combination of demographic, clinical and cellular analysis might help to identify possible monogenic cases. We have shown that in comparison with non-familial lupus, familial lupus patients present at an earlier age but in other respects are reasonably similar. We observed that familial lupus is more frequently associated with synovitis and a lower prevalence of dsDNA antibodies compared with non-familial cases, but there were no other significant differences in other clinical or serological features. We postulated that differences in familial lupus might arise from enrichment for MoFL cases, particularly when drawn from a population with a high rate of consanguinity. Indeed, within our cohort, the rate of consanguinity within the lupus familial lupus kindreds was ∼50%. The postulate that this might result in a higher rate of moFL was borne out. Although not all patients were available for sequencing, of those that were, we made a MoFL diagnosis in 36% cases. Analysis of the bona fide MoFL cases supports the contention that MoFL contributes to the overall differences between familial and non-familial lupus, as the differences observed in the MoFL cases were even more marked, with even younger age of presentation, specific clinical manifestations, and in some cases, distinct lymphocyte phenotypes. The implication is that these phenotypes might be used to help identify candidates for further genetic analysis within lupus clinics.

Our findings add to information available from other studies and have several implications. We identified a significant enrichment of monogenic lupus alleles. This is particularly noteworthy because our cohort was assembled from an adult lupus clinic, and included patients with adult-onset lupus. Thus, MoFL is not restricted to paediatric lupus. In our cohort, the median age of presentation in the familial lupus was 18 years *vs* 26 years for the non-familial cases. Other studies have shown a similar younger age of presentation in familial lupus in cohorts from populations with high rates of consanguinity. Thus, in a study from Kuwait, the mean age at onset of disease among the adult familial group was 20 years compared with 26.5 years for the non-familial cases [22]. By contrast, in studies from Finland and USA, where consanguinity is uncommon, age of presentation of familial SLE was 30 years and 36 years, respectively [15, 23].

In the present study, the most common clinical manifestations of familial MoFL, NonMoFL and non-familial adult Omani SLE were hematological manifestations: leukopenia and lymphopenia followed by synovitis. By contrast, other studies have identified different clinical manifestations. Synovitis and acute cutaneous lupus were the most common SLE clinical manifestations in a Romanian cohort [24], non-scarring alopecia in a Kuwaitian cohort [22] and leukopenia in a Finnish cohort [15] in both familial and non-familial groups. This difference in the prevalence of different clinical manifestations is likely due to the difference in the ethnicity of each of the described cohorts [25–27]. Moreover, previous studies have found no difference between the familial and non-familial SLE among the assessed clinical or immunological manifestations [15, 22, 28]. Contrary to this, we found that synovitis was more common in the familial compared with the non-familial SLE cases.

As expected, consanguinity appears to be a crucial factor predisposing to MoFL. By contrast, with our results, in a study of early-onset lupus within a population low rate of consanguinity, the rate of MoFL was much lower, even for early-onset lupus. Thus, in a cohort of 117 paediatric lupus cases (<16 y, median age 9 y) in which 20% had familial SLE, drawn from Europe, Asia and Africa, a diagnostic yield of 7% (eight moFL cases) were identified (*C1QA, C1QC, C2, DNAS1L3, IKZF1*). By design, all cases were enriched for young age, and the low rate of case ascertainment hampered identification of unique phenotypes [29]. In a small Isreali cohort (*n* = 15) of early onset (<18 years) lupus, six patients were sequenced and a diagnosis was made in four cases. Although distinguished phenotypically, this included non-lupus-related features such as dysmorphism and developmental delay and infection arising as part of the complex phenotype of the causal variants identified (*MAN2B1, SLC7A7, PTEN, STAT1*) [30].

Homozygosity for *DNASE1L3* was observed in consanguineous families. All shared the same allele, which was discovered in a previous Omani cohort, suggesting a founder effect for the relatively high prevalence of this form of lupus within Oman [20].We also identified AD forms of MoFL. For *IKZF2*, 8/8 sibs were heterozygous for the variant. While the mother is deceased and no material was available for genetic testing, if she were homozygous, we would expect this pattern of transmission, whereas if she were heterozygous, the chances of this transmission pattern are 1/2056. Thus, it is reasonable to infer that she was homozygous and this exemplifies how consanguinity may impact prevalence of AD disease.

In addition to early age of onset, there are some emerging phenotype-genotype relations with specific forms of MoFL. Thus, *TREX1* and other interefonopathy variants are associated with chilblain lupus and CNS involvement [31]. Patients with mutations associated with high inflammatory state such as *STAT1* gain-of-function, RAS-associated mutations and A20 haploinsufficiency present with oral ulcers [32]. Of the variants described here, *DNAS1L3* is associated with severe arthritis, nephritis as well as urticaria and hypocomplementaemia (HUVS) [33, 9, 34]. Indeed, it is possible that *DNASE1L3* variants are particularly associated with HUVS when it presents in childhood [35]. *COPA* variants are associated with interstitial lung disease and haemorrhage, arthritis and nephritis. So far, very few cases of *IKZF2* lupus have been described. Of note, our patients with *IKZF2* had presented with either primary or secondary anti-phospholipid syndrome with positive serology and thrombosis.

While a clinical diagnosis of lupus is made on the basis of clinical manifestations, autoimmune serology, and haematology, changes in circulating lymphocytes are often also present [36]. These include expansion of circulating follicular helper T cells [37] and CD21^low^ IgD- CD27- age-associated or atypical memory B cells [38].The latter have been observed more frequently in patients with lupus nephritis [39, 40]. Their number fluctuate with disease activity [41]. Our analysis of lymphocyte subsets among different MoFL cases identified some distinguishing features. Patients with variants in *DNASE1L3* exhibited a significant expansion of exhausted CD4+ T cells (PD-1+CD45RA+) compared with other MoFL cases and non-familial lupus.

Monogenic lupus is genetically heterogeneous, with >40 monogenic causes of lupus described to date (Supplementary Table S1, available at *Rheumatology* online). New causes continue to be identified. WES offers the opportunity to identify known causes, and once performed, genomes can be re-analysed periodically for new disease-associated variants. WGS offers the additional advantages of excellent coverage of all exons, and better detection of structural variants, although these benefits need to be weighed up against the increased cost of both sequencing and data storage [42]. Overall, the indications for WES and WGS in routine lupus clinics remains uncertain. Early age of onset and significant concurrent non-lupus phenotypes have been used to triage patients for sequencing. Our study is the first among adults from the Middle East region and is distinguished by the high proportion of consanguineous families that underwent exome sequencing, together with the use of lymphocyte analysis looking for cellular signatures. In the light of our results, WES also appears to be indicated in lupus cohorts drawn from populations with high rates of consanguinity irrespective of clinical manifestations or age of presentation.

Finally, it is important to highlight that our study was subject to the limitations of a retrospective analysis. Furthermore, it is possible that the clinical manifestations may differ in lupus cases referred to a tertiary centre. While recruitment of cases and controls from a single centre might have offset the risk of variation in environmental and lifestyle factors contributing to differences in phenotype, these were not characterized. For reasons outlined above, WGS rather than WES may have increased case ascertainment. The use of different diagnostic methods, in particular for assessment of dsDNA and phospholipid antibodies, over time might contribute to the guarded interpretation of such retrospective studies.

## Conclusion

The present study has shown that familial SLE presented earlier and was associated more with synovitis. In addition, among familial SLE, MoFL tend to present at a younger age than the NonMoFL and had more normalization of male-to-female ratio and had some cellular signature. Moreover, exome sequencing has helped in the diagnosis of around 36% of the recruited pedigrees.

## Supplementary material

Supplementary material is available at *Rheumatology* online.

## Supplementary Material

keaf304_Supplementary_Data

## Data Availability

Data are available on request to the authors.
