# Supplementary material for: Single-centre comparison of non-familial, familial and monogenic lupus
Source: Rheumatology (Oxford). 2025 Jun 4;64(10):5388–95. doi: 10.1093/rheumatology/keaf304 (PMC12494222; doi:10.1093/rheumatology/keaf304)
Supplement: keaf304_Supplementary_Data [file keaf304_supplementary_data.zip › keaf304_Supplementary_Data/rhe-25-0224-File003.docx]

**Supplementary Data S1**

**Familial systemic lupus erythematosus**

**Family A**

Patient 1 was diagnosed with SLE at the age of 4 years. She presented with a 2-month history of urticaria and joint pain due to active arthritis at both ankle joints. In 2010, she had pericardiocentesis for pericardial effusion. After cardiology review, she was managed with an increase in azathioprine and prednisolone. In 2012 her echocardiogram and MRI showed dilatation at aortic root, with no thickening of the wall of aorta to suggest aortitis. Her medication was changed to mycophenolate mofetil (MMF). In 2013, her inflammatory parameters were high without any evidence of organ-specific disease activity. She remained asymptomatic on MMF and hydroxychloroquine. In February 2019, she was admitted with pneumonia and responded well to antibiotics. After discharge, she was seen in the outpatient on March 2019 and at this time she remained well, however, shortly this visit, she was admitted to her local hospital and died. Cause of death was unknown.

Patient 2 was diagnosed with SLE at the age of 3 years. He presented with nephrotic syndrome and renal biopsy showed class IV lupus nephritis and positive SLE serology. He saw treated according to the NIH protocol with cyclophosphamide for 2 years, then with MMF. He continued to have flares of lupus nephritis despite MMF treatment, so was changed to rituximab, to which he responded very well. He remains well and asymptomatic, with normal renal function. His current medication is MMF, hydroxychloroquine, and irbesartan 150 mg once per day

Patient 3 was diagnosed with SLE at the age of 2 years. She presented with urticaria, fever, joint pain and anaemia. Her serology was consistent with SLE. Shortly afterwards she presented with renal impairment and had a renal biopsy showing class V lupus nephritis. She was treated with MMF and then with cyclophosphamide. In 2018 and 2019, she suffered with pericarditis that was managed with and increased dose of prednisolone. In July 2020, lung infiltrates were noted on high resolution CT scan, together with pleural and small pericardial effusions. A diagnosis of SLE-related ILD was made. She received cyclophosphamide, methylprednisolone pulses and IVIG. She further two episodes of pericarditis, each time treated with pulsed methylprednisolone, to which she responded well. A CT chest in May 2021 showed significant resolution of lung infiltrates, with mild residual atelectasis and bronchiectasis. She also had two later admissions with chest infection responding fully to antibiotics. Her most recent chest CT scan showed complete resolution of ground glass opacities and stable lower lobe bronchiectatic changes. Magnetic resonance imaging of her heart was normal. Her renal function remains normal.

**Family B**

Patient 4 was diagnosed with SLE at the age of six years when presented with urticaria, abdominal pain, joint pain and very low complement levels. Other than few episodes of urticaria, he remains well, with normal renal function.

Patient 5 was diagnosed with SLE at the age of six years after a 2-year history of urticaria, arthralgia, hypocomplementemia and a positive renal biopsy. She was managed initially with hydroxychloroquine then shifted to azathioprine, but her disease remained active and her renal function started to deteriorate. There were concerned regarding compliance with the treatment regimen, which was nevertheless modified to include rituximab was added to her regimen. She progressed to end-stage renal disease. She went on to receive a renal transplant and now remains well and asymptomatic.

**Family C**

Patient 6 was diagnosed with SLE at the age of six years after presenting with abdominal pain, urticaria, polyarthralgia and severe hypocomplementemia. Skin biopsy of the rask showed leukocytoclastic vasculitis. He presented once with pulmonary haemorrhage and was managed with methylprednisolone pulses and cyclophosphamide, then maintained on low-dose prednisolone and MMF. He had a few further disease flares in the form of abdominal pain, urticaria and joint pain, each managed with increased doses of oral prednisolone. He is currently well and asymptomatic.

Patient 7 was diagnosed with SLE at the age of 4 years after presenting with a very short history of abdominal pain, urticaria and joint pain. She was treated with prednisolone and azathioprine. She had recurrent episodes of abdominal pain, joint pains, urticaria, anaemia, and raised inflammatory parameters. High resolution chest CT scan showed ground glass changes and pulmonary function test showed impaired diffusion capacity. Azathioprine was replaced with MMF. Her respiratory disease progressed despite aggressive treatment. She represented with fever, cough, shortness of beath. Investigations revealed pericardial effusion and pulmonary haemorrhage. She was admitted to ICU for ventilatory support but died of respiratory failure.

**Family D**

Patient 8 was diagnosed with SLE and secondary phospholipid antibody syndrome at the age of 5 years. Throughout the course of his disease, he had multiple flares of the disease, mainly renal, and progressed to end-stage renal disease. He is currently on dialysis, awaiting renal transplant. In addition, at the age of 12 years, he suffered basal ganglia infarct and subsequently developed sensory neural hearing loss. At the age of 28 years was diagnosed with an atrial myxoma and possible atrial thrombus. He was managed with anticoagulation.

Patient 9 is currently 43 years old woman. She has a history of five spontaneous miscarriages. Investigations revealed high titres of phospholipid antibodies but ANA, antibodies to extractable nuclear antigens and dsDNA were not detected. She was therefore diagnosed with primary phospholipid antibody syndrome. During her subsequent pregnancies she was managed successfully with low molecular weight heparin and aspirin. She currently remains well.

Patient 10 was diagnosed with SLE at the age of 21 years. She presented with fever, arthritis and arthralgia, and cervical lymphadenopathy. Lymph node biopsy showed reactive follicular hyperplasia. Investigations revealed both positive lupus serology (strong ANA, strong positive nucleosome and PCNA on ENA as well as very strong dsDNA by ELISA) and phospholipid antibodies. She had one miscarriage and during two subsequent pregnancies she was managed successfully with anticoagulation.

**Family E**

Patient 11 is 36 years old. She was diagnosed with SLE at the age of 12 years. She presented with polyarthralgia, rash, Raynaud’s phenomenon and proteinuria. Investigations revealed ANA and dsDNA antibodies. Renal biopsy which showed lupus nephritis. She was treated with methylprednisolone pulses (no further details are available regarding her initial treatment). She had three episodes of venous thromboembolism. Two occurred while travelling. Tests for phospholipid antibodies have remained negative. Currently she is on long term anticoagulation. High resolution chest CT scan did not show any parenchymal lung disease. She remains well with normal renal function.

Patient 12 is 13 years old. She was diagnosed with SLE at the age of six years at another hospital. She presented with an 8-month history of polyarthritis and intermittent fever. Investigations revealed ANA, hypocomplementemia and leukopenia. Her current treatment includes belimumab, methotrexate and hydroxychloroquine. She currently remains well.
