# Supplementary material for: Single-centre comparison of non-familial, familial and monogenic lupus
Source: Rheumatology (Oxford). 2025 Jun 4;64(10):5388–95. doi: 10.1093/rheumatology/keaf304 (PMC12494222; doi:10.1093/rheumatology/keaf304)
Supplement: keaf304_Supplementary_Data [file keaf304_supplementary_data.zip › keaf304_Supplementary_Data/Supplementary_Tables_MU_FL.docx]

**Supplementary Table S1. Monogenic SLE**

| Gene*  (protein) | Inheritance | Additional Phenotype | References of original descriptions |
| --- | --- | --- | --- |
| *C1QA*  *C1QB*  *C1QC* | AR | Recurrent infection | [1] |
| *C1R*  *C1S* | AR | C1S deficiency, Ehlers-Danlos syndrome type 1 | [2, 3] |
| *C2* | AR | C2 deficiency | [4] |
| *C3* | AR | Pyogenic infection; membranoproliferative glomerulonephritis | [5] |
| *C4A*  *C4B* | AR | Pyogenic infection; HSP | [6] |
| *FCN3* | AR | Recurrent infection | [7] |
| *DNASE1* | AD |  | [8] |
| *DNASE1L3* | AR | HUVS | [9] |
| *DNASE2* | AR | Early-onset liver fibrosis and systemic autoinflammatory manifestations and pancytopenia | [10] |
| *TREX1* | AD | Aicardi-Goutieres syndrome (AGS), Chilblain lupus, SSd | [11, 12] |
| *RNASEH2A*  *RNASRH2B*  *RNASRH2C* | AR | AGS | [13] |
| *SAMHD1* | AR | AGS; Chilblain lupus | [14] |
| *ADA2*  *(CECR1)* |  | Vasculitis, autoinflammation, cytopenia, Sneddon syndrome | [15] |
| *SAT1* | X-linked | NA | [16] |
| *RIG1*  (*DDX58)* | AD | Short stature, glaucoma, aortic calcification (Singleton-Merten syndrome 2) | [17, 18] |
| *STING1*  (TMEM173) | AD | Sting-associated vasculopathy, infantile onset (SAVI);  Chillblain lupus | [19, 20] |
| *IFIH1*  (MDA5) | AD | Short stature, glaucoma, aortic calcification, heart failure (Singleton-Merten syndrome 1); AGS | [21, 22] |
| *TLR7* | XLD | Transverse myelitis; Neuromyelitis optica | [23] |
| *UNC93B1* | AD |  | [24-26] |
| *IKZF1*  (IKAROS) | AD | CVID, ITP | [27] |
| *IKZF2*  (HELIOS) | AD | Immune deficiency | [28] |
| *TNFAIP3*  (A20) | AD | Autoinflammatory syndrome, Behçet-like; organ-specific autoimmunity | [29] |
| *ACP5*  (TRAP) | AR | Spondyloenchondrodysplasia with immune dysregulation (SPENCD) | [30] |
| *PACSIN1* | AD |  | [31] |
| *NLRC4* | AD | Familial cold autoinflammatory syndrome, Autoinflammation with infantile enterocolitis | [32] |
| *ADAR* | AR | AGS | [33] |
| *ISG15* | AR | Basal ganglia calcification; Mendelian susceptibility to mycobacteria; AGS | [34] |
| *STAT1* | AD | Chronic mucocutaneous candidiasis and autoimmune hypothyroidism | [35] |
| *OTUD1* | AD | Autoimmune disorders | [36] |
| SBDS | AR | Exocrine pancreas insufficiency, Shwachman-Bodian-Diamond Syndrome | [37] |
| *FASLG* | AD | ALPS | [38] |
| *PRKCD*  (PKCδ) | AR | ALPS, recurrent infection | [39, 40] |
| *KRAS*  *SHOC2*  *PTPN11* | AD | Noonan syndrome; cardiac defects and craniofacial dysmorphia  (RASopathy**)** | [41, 42]  [43] |
| *PIK3CD*  (PI3K; p110δ) | AD | Combined Immunodeficiency | [44] |
| *DOCK8* | AR | Hyper-IgE syndrome with recurrent infections | [45] |
| *SH2B3* |  | Thrombocythemia | [46] |
| *P2RY8* |  | NA | [47] |
| *BACH2* | AD | Immunodeficiency and autoimmunity | [48] |
| *LRBA* | AR | CVID, immunedysregulation | [49] |
| *RAG2* | AD | Granulomas | [50] |
| *CYBB*  (NOX2) | X-linked | Chronic granulomatous disease | [51] |
| *SLC7A7* | AR | Obesity, hyperammonemia, alveolar proteinosis | [52] |
| *PEPD*  (Prolidase) | AR | Developmental delay, proptosis, hyper IgE, dermatitis (prolidase deficiency) | [53] |
| *MAN2B1*  *(LAMAN)* | AR | Dysostosis, retinal degeneration, hypertrichosis, developmental delay | [54] |

*Approved gene name with alternative gene name (capitalised italics), and protein name (non-italicised) where appropriate. AGS, Aicardi-Goutieres syndrome; ALPS, Autoimmune lymphoproliferative syndrome; CVID, common variable immune deficiency; HSP, Henoch-Schoenlein purpura; HUVS, hypocomplementaemic urticarial vasculitis syndrome; ITP, Immune-mediated thrombocytopenic purpura; SSd, Sjogren’s syndrome.

**References**

1 Wara DW, Reiter EO, Doyle NE, Gewurz H, Ammann AJ. Persistent Clq deficiency in a patient with a systemic lupus erythematosus-like syndrome. The Journal of Pediatrics 1975;86(5):743-5.

2 Pondman KW, Stoop JW, Cormane RH, Hannema AJ. Abstracts of Papers Presented at the Third International Complement Workshop, Harvard Medical School, Boston, Massachusetts, June 3–5, 1968: Abnormal C′1 in a Patient with Systemic Lupus Erythematosus. The Journal of Immunology 1968;101(4):811-.

3 Suzuki Y, Ogura Y, Otsubo O, Akagi K, Fujita T. Selective deficiency of C1s associated with a systemic lupus erythematosus-like syndrome. Report of a case. Arthritis Rheum 1992;35(5):576-9.

4 Klemperer MR, Woodworth HC, Rosen FS, Austen KF. Hereditary deficiency of the second component of complement (C'2) in man. J Clin Invest 1966;45(6):880-90.

5 McLean RH, Lowenstein M, Rothfield N. HETEROZYGOUS C3 DEFICIENCY ASSOCIATED WITH A SYSTEMIC LUPUS ERYTHEMATOSUS-LIKE DISEASE. Pediatric Research 1977;11(4):490-.

6 Fielder AH, Walport MJ, Batchelor JR, et al. Family study of the major histocompatibility complex in patients with systemic lupus erythematosus: importance of null alleles of C4A and C4B in determining disease susceptibility. Br Med J (Clin Res Ed) 1983;286(6363):425-8.

7 Troldborg A, Steffensen R, Trendelenburg M, et al. Ficolin-3 Deficiency Is Associated with Disease and an Increased Risk of Systemic Lupus Erythematosus. Journal of Clinical Immunology 2019;39(4):421-9.

8 Yasutomo K, Horiuchi T, Kagami S, et al. Mutation of DNASE1 in people with systemic lupus erythematosus. Nat Genet 2001;28(4):313-4.

9 Al-Mayouf SM, Sunker A, Abdwani R, et al. Loss-of-function variant in DNASE1L3 causes a familial form of systemic lupus erythematosus. Nature Genetics 2011;43(12):1186-8.

10 Rodero MP, Tesser A, Bartok E, et al. Type I interferon-mediated autoinflammation due to DNase II deficiency. Nat Commun 2017;8(1):2176.

11 Lee-Kirsch MA, Chowdhury D, Harvey S, et al. A mutation in TREX1 that impairs susceptibility to granzyme A-mediated cell death underlies familial chilblain lupus. J Mol Med (Berl) 2007;85(5):531-7.

12 Lee-Kirsch MA, Gong M, Chowdhury D, et al. Mutations in the gene encoding the 3'-5' DNA exonuclease TREX1 are associated with systemic lupus erythematosus. Nat Genet 2007;39(9):1065-7.

13 Günther C, Kind B, Reijns MAM, et al. Defective removal of ribonucleotides from DNA promotes systemic lupus erythematosus. Pediatric Rheumatology 2015;13(1):O86.

14 Ramantani G, Häusler M, Niggemann P, et al. Aicardi-Goutières Syndrome and Systemic Lupus Erythematosus (SLE) in a 12-Year-Old Boy With SAMHD1 Mutations. Journal of Child Neurology 2011;26(11):1425-8.

15 Schepp J, Bulashevska A, Mannhardt-Laakmann W, et al. Deficiency of Adenosine Deaminase 2 Causes Antibody Deficiency. J Clin Immunol 2016;36(3):179-86.

16 Xu L, Zhao J, Sun Q, et al. Loss-of-function variants in <em>SAT1</em> cause X-linked childhood-onset systemic lupus erythematosus. Annals of the Rheumatic Diseases 2022;81(12):1712-21.

17 Peng J, Wang Y, Han X, et al. Clinical Implications of a New DDX58 Pathogenic Variant That Causes Lupus Nephritis due to RIG-I Hyperactivation. J Am Soc Nephrol 2023;34(2):258-72.

18 Raupov R, Suspitsin E, Belozerov K, Gabrusskaya T, Kostik M. IFIH1 and DDX58 gene variants in pediatric rheumatic diseases. World J Clin Pediatr 2023;12(3):107-14.

19 Jeremiah N, Neven B, Gentili M, et al. Inherited STING-activating mutation underlies a familial inflammatory syndrome with lupus-like manifestations. J Clin Invest 2014;124(12):5516-20.

20 König N, Fiehn C, Wolf C, et al. Familial chilblain lupus due to a gain-of-function mutation in STING. Ann Rheum Dis 2017;76(2):468-72.

21 Rice GI, del Toro Duany Y, Jenkinson EM, et al. Gain-of-function mutations in IFIH1 cause a spectrum of human disease phenotypes associated with upregulated type I interferon signaling. Nature Genetics 2014;46(5):503-9.

22 Van Eyck L, De Somer L, Pombal D, et al. Brief Report: IFIH1 Mutation Causes Systemic Lupus Erythematosus With Selective IgA Deficiency. Arthritis Rheumatol 2015;67(6):1592-7.

23 Brown GJ, Cañete PF, Wang H, et al. TLR7 gain-of-function genetic variation causes human lupus. Nature 2022;605(7909):349-56.

24 David C, Arango-Franco CA, Badonyi M, et al. Gain-of-function human UNC93B1 variants cause systemic lupus erythematosus and chilblain lupus. J Exp Med 2024;221(8).

25 Wolf C, Lim EL, Mokhtari M, et al. UNC93B1 variants underlie TLR7-dependent autoimmunity. Sci Immunol 2024;9(92):eadi9769.

26 Al-Azab M, Idiiatullina E, Liu Z, et al. Genetic variants in UNC93B1 predispose to childhood-onset systemic lupus erythematosus. Nat Immunol 2024;25(6):969-80.

27 Hoshino A, Okada S, Yoshida K, et al. Abnormal hematopoiesis and autoimmunity in human subjects with germline <em>IKZF1</em> mutations. Journal of Allergy and Clinical Immunology 2017;140(1):223-31.

28 Shahin T, Mayr D, Shoeb MR, et al. Identification of germline monoallelic mutations in IKZF2 in patients with immune dysregulation. Blood Adv 2022;6(7):2444-51.

29 Aeschlimann FA, Batu ED, Canna SW, et al. A20 haploinsufficiency (HA20): clinical phenotypes and disease course of patients with a newly recognised NF-kB-mediated autoinflammatory disease. Ann Rheum Dis 2018;77(5):728-35.

30 Briggs TA, Rice GI, Daly S, et al. Tartrate-resistant acid phosphatase deficiency causes a bone dysplasia with autoimmunity and a type I interferon expression signature. Nat Genet 2011;43(2):127-31.

31 Xie C, Zhou H, Athanasopoulos V, et al. De Novo Gene Variant Found in Childhood Lupus and a Role for PACSIN1/TRAF4 Complex in Toll-like Receptor 7 Activation. Arthritis & Rheumatology 2023;75(6):1058-71.

32 Wang Q, Ye X, Zheng W, et al. NLRC4 gain-of-function variant is identified in a patient with systemic lupus erythematosus. Clin Immunol 2023;255:109731.

33 Crow YJ, Chase DS, Lowenstein Schmidt J, et al. Characterization of human disease phenotypes associated with mutations in TREX1, RNASEH2A, RNASEH2B, RNASEH2C, SAMHD1, ADAR, and IFIH1. Am J Med Genet A 2015;167a(2):296-312.

34 Al-Mayouf SM, Akbar L, AlEnazi A, Al-Mousa H. Autosomal Recessive ISG15 Deficiency Underlies Type I Interferonopathy with Systemic Lupus Erythematosus and Inflammatory Myositis. Journal of Clinical Immunology 2021;41(6):1361-4.

35 Toubiana J, Okada S, Hiller J, et al. Heterozygous STAT1 gain-of-function mutations underlie an unexpectedly broad clinical phenotype. Blood 2016;127(25):3154-64.

36 Lu D, Song J, Sun Y, et al. Mutations of deubiquitinase OTUD1 are associated with autoimmune disorders. J Autoimmun 2018;94:156-65.

37 Zhang T, Yu Z, Gao S, Wang L, Song H. Systemic Lupus Erythematosus in Shwachman-Diamond Syndrome: a Novel Phenotype. J Clin Immunol 2023;43(3):550-3.

38 Wu J, Wilson J, He J, Xiang L, Schur PH, Mountz JD. Fas ligand mutation in a patient with systemic lupus erythematosus and lymphoproliferative disease. J Clin Invest 1996;98(5):1107-13.

39 Belot A, Kasher PR, Trotter EW, et al. Protein Kinase Cδ Deficiency Causes Mendelian Systemic Lupus Erythematosus With B Cell-Defective Apoptosis and Hyperproliferation. Arthritis & Rheumatism 2013;65(8):2161-71.

40 Jefferson L, Ramanan AV, Jolles S, et al. Phenotypic Variability in PRKCD: a Review of the Literature. J Clin Immunol 2023;43(8):1692-705.

41 Leventopoulos G, Denayer E, Makrythanasis P, Papapolychroniou C, Fryssira H. Noonan syndrome and systemic lupus erythematosus in a patient with a novel KRAS mutation. Clin Exp Rheumatol 2010;28(4):556-7.

42 Bader-Meunier B, Cavé H, Jeremiah N, et al. Are RASopathies new monogenic predisposing conditions to the development of systemic lupus erythematosus? Case report and systematic review of the literature. Seminars in Arthritis and Rheumatism 2013;43(2):217-9.

43 Quaio CRDC, Carvalho JF, da Silva CA, et al. Autoimmune disease and multiple autoantibodies in 42 patients with RASopathies. American Journal of Medical Genetics Part A 2012;158A(5):1077-82.

44 Li GM, Liu HM, Guan WZ, et al. A mutation in PIK3CD gene causing pediatric systemic lupus erythematosus: A case report. Medicine 2019;98(18):e15329.

45 Jouhadi Z, Khadir K, Ailal F, et al. Ten-Year Follow-Up of a DOCK8-Deficient Child With Features of Systemic Lupus Erythematosus. Pediatrics 2014;134(5):e1458-e63.

46 Zhang Y, Morris R, Brown GJ, et al. Rare SH2B3 coding variants in lupus patients impair B cell tolerance and predispose to autoimmunity. J Exp Med 2024;221(4).

47 He Y, Gallman AE, Xie C, et al. P2RY8 variants in lupus patients uncover a role for the receptor in immunological tolerance. J Exp Med 2022;219(1).

48 Zhou L, Sun G, Chen R, et al. An early-onset SLE patient with a novel paternal inherited BACH2 mutation. J Clin Immunol 2023;43(6):1367-78.

49 Liphaus BL, Caramalho I, Rangel-Santos A, Silva CA, Demengeot J, Carneiro-Sampaio MMS. LRBA deficiency: a new genetic cause of monogenic lupus. Ann Rheum Dis 2020;79(3):427-8.

50 Walter JE, Lo MS, Kis-Toth K, et al. Impaired receptor editing and heterozygous RAG2 mutation in a patient with systemic lupus erythematosus and erosive arthritis. Journal of Allergy and Clinical Immunology 2015;135(1):272-3.

51 Schmitt CP, Schärer K, Waldherr R, Seger RA, Debatin KM. Glomerulonephritis associated with chronic granulomatous disease and systemic lupus erythematosus. Nephrol Dial Transplant 1995;10(6):891-5.

52 Bissonnette R, Friedmann D, Giroux J-M, et al. Prolidase deficiency: A multisystemic hereditary disorder. Journal of the American Academy of Dermatology 1993;29(5, Part 2):818-21.

53 Kurien BT, D'Sousa A, Bruner BF, et al. Prolidase deficiency breaks tolerance to lupus-associated antigens. Int J Rheum Dis 2013;16(6):674-80.

54 Urushihara M, Kagami S, Yasutomo K, et al. Sisters with α-mannosidosis and systemic lupus erythematosus. European Journal of Pediatrics 2004;163(4-5):192-5.

**Supplementary Table S2.** Primers.

| Gene | Variant | Primer | Primer sequence (5`→3′) |
| --- | --- | --- | --- |
| *IKZF2*  (NM_016260.3) | c.1536T>G  p.(Tyr512Ter) | F | GGGCTCTCTGAAGGACATCT |
|  |  | R | AGGCAGAGCAAATGACACTG |
| *COPA*  (NM_004371.4) | c.2294A>G  p.(His765Arg) | F | TGAATACCAGGAGTCAGGGA |
|  |  | R | AGGTGGGAGAGTGACATTCA |
| *DNASE1L3* (NM_004944.4) | c.643del  p.(Trp215GlyfsTer2) | F | TTGTTTACTGGGCCTGGGAC |
|  |  | R | ATCGAGGCCTCCCAAAGTG |

**Supplementary Table S3.** Cell subset analysis.

| Cell type | Phenotype | p value^1^ | Adjusted p value^2^ |
| --- | --- | --- | --- |
| B cells | CD19+ | 0.10 | 0.32 |
| Transitional B cells | CD24+ CD38+ | 0.35 | 0.51 |
| Atypical B cells | CD19+CD21^low^ | 0.15 | 0.34 |
| Marginal zone B cells | IgD+IgM+ | 6.6x10^-3^ | 0.055 |
| Naïve B cells | IgD+ IgM^low^ CD27- | 0.15 | 0.34 |
| Memory B cells |  | 0.033 | 0.15 |
| IgM+ memory B cells | CD27+ IgD- IgM+ | 0.47 | 0.66 |
| IgD+ memory B cells | CD27+ IgD+ IgM- | 7.9x10^-3^ | 0.055 |
| IgD+ IgM+ memory B cells | CD27+ IgD+ IgM+ | 0.55 | 0.70 |
| Switched memory B cells | CD27+ IgD- IgM- | 0.17 | 0.34 |
| Double negative B cells | CD27- IgD- | 0.045 | 0.16 |
| T cells | CD3+ | 0.16 | 0.34 |
| CD4+ T cells | CD3+ CD4+ | 0.89 | 0.91 |
| Naïve CD4+ T cells | CD3+ CD4+ CCR7+ CD45RA+ | 0.015 | 0.084 |
| Central memory CD4+ T cells | CD3+ CD4+ CCR7+ CD45RA- | 0.12 | 0.34 |
| Effector memory CD4+ T cells | CD3+ CD4+ CCR7- CD45RA+ | 0.53 | 0.70 |
| CD4+ TEMRA | CD3+ CD4+ CCR7- CD45RA+ | 0.20 | 0.37 |
| Exhausted CD4+ T cells (a) | CD4+ CD279+ CD45RA- | 0.043 | 0.16 |
| Exhausted CD4+ T cells (b) | CD4+ CD279+ CD45RA+ | **1.0x10^-5^** | **1.7x10^-4^** |
| CD8+ T cells | CD3+ CD8+ | 0.88 | 0.91 |
| Naïve CD8+ T cells | CD3+ CD8+ CCR7+ CD45RA+ | 0.31 | 0.50 |
| Central memory CD8+ T cells | CD3+ CD8+ CCR7+ CD45RA- | 0.84 | 0.91 |
| Effector memory CD8+ T cells | CD3+ CD8+ CCR7- CD45RA+ | 0.32 | 0.50 |
| CD8+ TEMRA | CD3+ CD4+ CCR7- CD45RA+ | 0.91 | 0.91 |
| Exhausted CD8+ T cells (a) | CD8+ CD279+ CD45RA- | 0.57 | 0.70 |
| Exhausted CD8+ T cells (b) | CD8+ CD279+ CD45RA+ | 5.73x10^-3^ | 0.055 |
| Central memory CD8+ T cells | CD3+ CD8+ CCR7+ CD45RA+ | 0.23 | 0.41 |
| Effector memory CD8+ T cells | CD3+ CD8+ CCR7+ CD45RA- | 0.67 | 0.79 |

^1^ANOVA; ^2^FDR correction for multiple tests
